# Improving the accuracy of a solid spherical source radius and depth estimation using the diffusion equation in fluorescence reflectance mode

**DOI:** 10.1186/1475-925X-9-28

**Published:** 2010-06-19

**Authors:** Marjaneh Hejazi, Florian Stuker, Divya Vats, Markus Rudin

**Affiliations:** 1 Medical Physics and Biomedical Engineering Department, School of Medicine, Tehran University of Medical Sciences, 1417613151 Tehran, Iran; 2 Research Center for Science and Technology in Medicine, Tehran University of Medical Sciences, Imam Khomeini Hospital, Keshavarz Blvd, 1417613151 Tehran, Iran; 3 Institute for Biomedical Engineering, University and ETH Zurich, Wolfgang-Pauli-Str. 10, 8093 Zurich, Switzerland; 4 Institute of Pharmacology and Toxicology, University Zürich, Winterthurerstr. 190, 8057 Zurich, Switzerland

## Abstract

**Background:**

Non-invasive planar fluorescence reflectance imaging (FRI) is used for accessing physiological and molecular processes in biological tissue. This method is efficiently used to detect superficial fluorescent inclusions. FRI is based on recording the spatial radiance distribution (SRD) at the surface of a sample. SRD provides information for measuring structural parameters of a fluorescent source (such as radius and depth). The aim of this article is to estimate the depth and radius of the source distribution from SRD, measured at the sample surface. For this reason, a theoretical expression for the SRD at the surface of a turbid sample arising from a spherical light source embedded in the sample, was derived using a steady-state solution of the diffusion equation with an appropriate boundary condition.

**Methods:**

The SRD was approximated by solving the diffusion equation in an infinite homogeneous medium with solid spherical sources in cylindrical geometry. Theoretical predications were verified by experiments with fluorescent sources of radius 2-6 mm embedded at depths of 2-4 mm in a tissue-like phantom.

**Results:**

The experimental data were compared with the theoretical values which shows that the root mean square (RMS) error in depth measurement for nominal depth values d = 2, 2.5, 3, 3.5, 4 mm amounted to 17%, 5%, 2%, 1% and 5% respectively. Therefore, the average error in depth estimation was ≤ 4% for depths larger than the photon mean free path.

**Conclusions:**

An algorithm is proposed that allows estimation of the location and radius of a spherical source in a homogeneous tissue-like phantom by accounting for anisotropic light scattering effect using FRI modality. Surface SRD measurement enabled accurate estimates of fluorescent depth and radius in FRI modality, and can be used as an element of a more general tomography reconstruction algorithm.

## Background

Optical imaging methods such as fluorescence reflectance imaging (FRI), bioluminescent imaging and fluorescent tomography are used both in the areas of clinical research, such as dermatological and intra-operative imaging and in pre-clinical research for small animal imaging [1]. A conventional FRI system records the spatial distribution of the scattered fluorescent light at the surface of a sample. This light distribution contains sufficient information for determining structural parameters such as radius and depth of fluorescence probe within biological tissue assuming a distribution of spherical sources. Information on depth and radius is required for deriving quantitative information on the source intensity, which may be translated into information of molecular and cellular processes within the tissue.

Recently, methods of the depth reconstruction for fluorescence molecular tomography have been widely studied [2-4]. In transmission mode, D'Andrea et al. [5] reported an analytical algorithm to recover the coordinates of a fluorescent inclusion embedded in a turbid medium with accuracy better than 2 mm. Kuo et al.[6] developed a three dimensional reconstruction method for localizing and determining the photon flux of the sources only from a single view. The experimental evaluation revealed that the average error in depth reconstruction for a point source in a sample tissue was 3% for depth larger than 7 mm and exceeded 50% for the superficial depths (≤ 2.5 mm) [7]. The lack of reconstruction accuracy was attributed to limitations in the assumption that photons were scattered isotropically in biological tissue. Eisdath et al. [8] proposed a theoretical model of photon migration in reflectance mode for localizing the fluorescent inclusion deeply embedded in a turbid media. The source coordinates were obtained with an average error of 4% at 3 mm and less than 10% for depths larger than 6 mm. Up to our knowledge, the radius estimation for superficial fluorescent inclusions in tissue like phantoms has not been reported in FRI modality to date.

The objective of this article is to improve the accuracy of the estimation of source depth and radius in reflection mode by accounting for anisotropic photon scattering. The proposed algorithm can also be regarded as important part of tomographic reconstruction algorithms.

## Methods

### Theoretical Background

The intensity distribution at the surface of a turbid medium is described by a spatial radiance distribution (SRD). This SRD was estimated by solving the diffusion equation in an infinite homogeneous medium with solid spherical sources of radius *R *with its center located at depth *z*.

**Figure 1:**
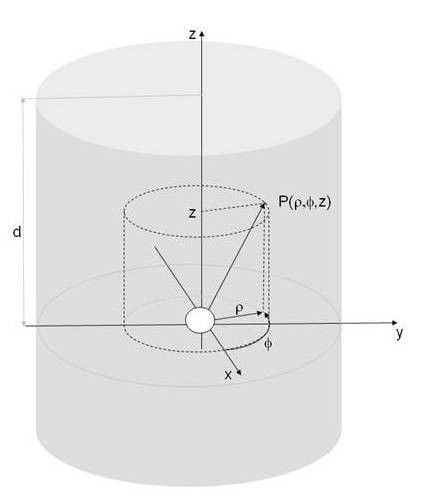
**Theoretical model**: A spherical light source of radius R is located at the origin of the coordinate system in a slab of thickness d. For the derivation of the SRD a cylindrical coordinatesystem ρ,φ, z has been used.

For deriving the theoretical SRD, an optical imaging system with the geometry depicted in Fig. 1 was chosen. It consisted of a slab of thickness d characterized by an absorption coefficient *μ*_*a *_and a reduced scattering coefficient , both with values corresponding to biological tissue.

A spherical light source is placed at the origin of a cylindrical coordinate system ρ, ϕ z. The SRD in a highly scattering medium is obtained from solving the diffusion equation for photon propagation [9,10]:(1)

where Φ(**r**) is the photon fluence (photons/cm^2^) at location **r **with the radius given by  for *z *≤ *d*, Ω describes the region of interest, *S*(**r**) an isotropic source term, while the diffusion coefficient *D*(**r**) = 3(*μ*_*a*_(**r**) + *μ*_*s*_'(**r**)), accounts for diffusive photon propagation. This equation is derived from the adiative transfer equation [11] by applying the diffusion approximation and is valid for highly scattering media of a thickness greater than the photon mean-free path length.

Recently, the steady-state solution of the diffusion equation with a solid spherical source of radius *R *embedded in an infinite homogenous medium has been formulated as [12]:(2)

with

where *P *is the source power  denotes the effective attenuation coefficient, while *α*(*R*) stands for the exponential terms given in brackets.

Φ(**r**, *R*) at the interface between two media with different refractive indices can be approximated by an index-mismatched Robin-type boundary condition. This condition for Φ(**r**, *R*) on ∂Ω is described in the following formula [13,14]:(3)

The term **n **is the unit vector normal to a surface element ∂Ω and *A *is a parameter governing the internal reflection at the boundary. The value of *A *= (1+R)/(1-R) depends on the relative refractive index mismatch between tissue and air and can be derived from Fresnel's law, with R as [14]:(4)

and *n *= *n*_*a*_/*n*_*t*_, *n_a _*and *n_t _*being the refractive indices for air and tissue, respectively. This equation yields *A *= 2.51 for the air-phantom interface with typical value of *n *= 1.33 [15]. Based on the boundary condition the measured fluence rate on ∂Ω is [13,16].(5)

Φ(**r**, *R*) can be converted to the radiance  for a perfectly isotropic light distribution:(6)

Because the radiance in biological tissue is slightly anisotropic and angle dependent due to the large number of scattering events, an angular dependent radiance *L*(**r**, *R*, ) is introduced which accounts for anisotropic optical properties of the tissue at **r **in direction of , i.e the expression for the radiance (Eq. (6)) is expanded according to [17]:(7)

with the second term denoting the net energy flux **F**(**r**) = -*D*(**r**)▽Φ(**r**, *R*) at a distance r from the sphere in direction [16]. Combining the various equations one obtains:(8)

which for **r **∈ *S *corresponds to the radiance distribution at the surface *S *of the sample that is generated by a spherical light source of radius *R*, i.e to the SRD.

### Experimental Evaluation

**Figure 2:**
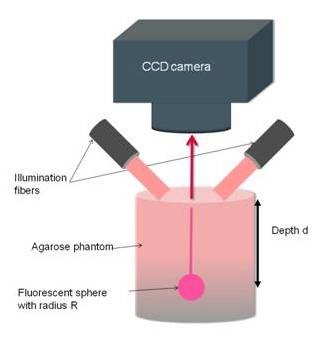
**Geometry of experimental setup for determining the SRD using fluorescence imaging in reflection mode**.

For experimental evaluation, a commercial in-vivo fluorescence imaging system (Maestro, Cri Woburn, USA) has been used with a geometry depicted in Fig. 2. The system consists of a fiber-delivered Xenon excitation source (*λ *= 500-950 nm, 30 W), a standard excitation and emission filter set and in addition a liquid crystal tunable filter (LCTF). The LCTF transmits a narrow bandwidth (10 nm) of the emitted fluorescence light to a 1.3 megapixel CCD camera. A 615-665 nm excitation filter and a 700 nm long-pass emission filter were used for the evaluation.

The phantom was made from 6 g agarose (BioGene, Kimbolton, UK), 24 ml Intralipid 20% (Fresenius SE, Bad Homburg, Germany) and 18 μl Indian ink (Pelikan Holding, Schindellegi, Switzerland) [18] dissolved in 600 ml water. The optical properties of the phantom (with radius 10 cm) were measured with a frequency-domain tissue oximeter (oxiplexTS, ISS, Champaign, USA). The reduced scattering and absorption coefficients, at 692 nm, were then found to be  = 8 cm^-1 ^and μ_a _= 0.11 cm^-1 ^respectively. The same mixture was poured into four plexiglass molds of 6 × 6 cm and thickness ranging from 2 mm to 4 mm. The width of the mould was more than 10 penetration depths (0.6 cm) which fulfilled the requirement for a semi-infinite boundary medium [19]. After solidification, the agarose layers were removed from the molds and positioned by a fixed holder on the top of solid spherical source. The sources with radius 2-6 mm contained 5 mg/l near-infrared quantum dot (Qdot 705 ITK, Invitrogen, Basel, Switzerland) with emission wavelength at 705 nm.

The optical images were recorded for varying thickness *d *and denoised by a bandpass filter [20]. The bandpass filter consists of a Gaussian low-pass filter and a boxcar kernel which was used as a high-pass filter. First, a low pass filtered image was generated by convolving the diffuse image with the Gaussian filter, a high-pass filtered image was then obtained by convolving the diffuse image with the boxcar function. The difference between low-pass filtered image and high-pass filtered image allowed extraction of the final image for quantitative analysis.

## Results

**Figure 3:**
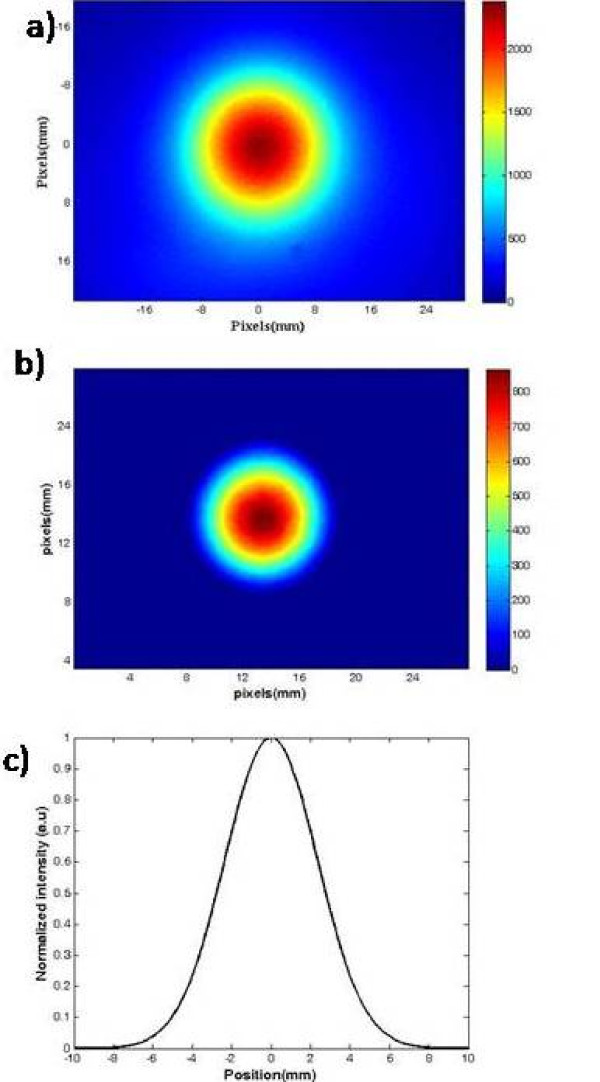
**a) The diffuse image of a QD source of radius 6 mm in the depth of 3.5 mm at 620 nm**. b) The SRD was extracted by using the bandpass filter. c) The full width at half maximum (FWHM) of the SRD was obtained from the filtered SRD using Gaussian fitting.

Fig. 3a displays the intensity distribution at the sample surface for a spherical quantum dot source of radius 6 mm located at d = 3.5 mm. For excitation and detection bandpass filters have been used. The original image was filtered using the bandpass filter (Fig. 3b). The full width at half maximum (FWHM) of intensity distribution (SRD) was derived using Gaussian fitting and amounted to 5.5 mm (Fig. 3c).

**Figure 4:**
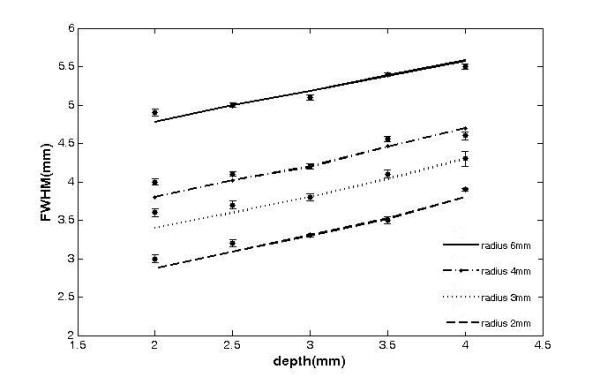
**a) FWHM as a function of the depth for the fluorescent sources of radius 2-6 mm located in a highly turbid media**. The experimental and the theoretical data are represented by lines and solid dots respectively. The error bars denote the standard deviation of five measurements.

**Figure 5:**
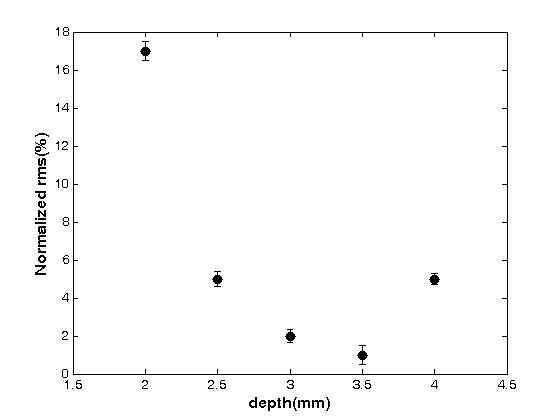
**RMS error of the depth reconstruction as function of depth (mm) for source radii R = 2 to 6 mm**. The normalized RMS was obtained as  with *d_exp, i _*and *d*_0 _being the experimental and nominal depths, respectively, where *N *is the number of measurements.

The experimental FWHM data were compared with the theoretical values obtained as FWHM of L(**r**, R)(Eq. (5)) as function of d and radius (Fig. 4). Source radii range from 2 mm to 6 mm, while d was varied between 2 mm and 4 mm. In general, good agreement between experimental and theoretical FWHM values has been found. Error estimation revealed that the RMS of depth measurement errors for depth values d = 2, 2.5, 3, 3.5, 4 mm were 17%, 5%, 2%, 1% and 5% respectively, for the source radii 2-6 mm covered in our experiment (Fig. 5).

## Discussion and Conclusions

In this work, we have shown that the structural parameters of a spherical source (depth and radius) can be estimated by measuring the spatial radiance distribution (SRD) at the sample surface. The SRD at the surface of a turbid medium was derived by calculating a steady-state solution of the diffusion equation using the Robin boundary condition.

The accuracy of the theoretical model was validated by fluorescence reflectance imaging of a hemispace homogeneous phantom containing fluorescent source. The comparison showed the RMS of depth measurement errors for depth values d = 2, 2.5, 3, 3.5, 4 mm were 17%, 5%, 2%, 1% and 5% respectively. As discussed in previous studies, the relatively large (17%) overestimation at a depth of 2 mm was attributed to the inaccuracy of the diffusion approximation for distances comparable to the mean free path (1/( + μ_a_) of the photon propagation in a turbid medium [15]. For depth values exceeding 4 mm the decrease in fluorescence intensity at the sample surface became a limiting factor; as a consequence of decreased signal-to-noise ratios extraction of accurate SRDs using the denoising method described became difficult and significant errors in FWHM estimation arose. Comparing our method with the procedure described earlier [6] we obtained improved depth estimates close to the sample surface (5% versus 45% average error). This might indicate the importance of accounting for the anisotropic distribution of the scattered light in highly turbid media when deriving the SRD, in particular when analyzing source locations within a few millimeter from the sample surface. Eisdath et al. [8] have developed a model for reflectance mode imaging for localizing a fluorescent source in three dimensions. Their results showed that the average error in source localization was less than 10% for depths larger than 6 mm. However, when using a fluorescence reflectance system SNR becomes a limiting factor for deriving accurate information on source position and dimension within a turbid medium for depth values large than 5 mm.

The algorithm in its current form is not suited for the accurate determination of source location and radius in irregular biological tissues, in particular when considering reconstruction from a single view. The major problem when translating the model to an irregular and heterogeneous sample is that the solution of the inverse problem for the fluorescent source structural parameter does not yield a unique SRD from measurements at a single wavelength [4,6]. Next steps in the model development are therefore extension to multiple wavelengths, which will improve the accuracy of the reconstructed source distributions [4,21]. A multi-wavelength algorithm can recover the source depth and radius due to the wavelength dependence of SRD (Eqs. (3) and (5)).

In conclusion, an algorithm is proposed that allows estimation of the location and radius of a spherical source in a homogeneous tissue-like phantom by accounting for anisotropic light scattering effect using FRI modality. Experimental validation revealed the accuracy and limitations of the current model. It is obvious that heterogeneity of optical parameters as found in biological tissues will affect the accuracy of the source reconstruction algorithm. These effects will be analyzed in future work.

## Competing interests

The authors declare that they have no competing interests.

## Authors' contributions

MH and FS participated in the design of the study, derived the theoretical formula, performed the experimentscarried, drafted and revised the manuscript. DV participated in the experimental evaluation and helped to revise the manuscript. MR participated in the theoretical study design, supervised the experimentd and revised the manuscript. All authors read and approved the final manuscript.
